# Association Between Gut-Brain Axis Dysfunction and Vestibular Migraine Severity

**DOI:** 10.7759/cureus.91392

**Published:** 2025-09-01

**Authors:** Muhammad Alryan Masood, Namra Asif, Muhibullah Younus, Bhavna Singla, Sheraz Khan, Shivam Singla, Syed Hassan Elahi, Nazia Parveen, Muhammad Ahsan Ishfaq, Tehmina Jamil

**Affiliations:** 1 Stroke Medicine, University Hospital of Wales, Cardiff, GBR; 2 Internal Medicine, Mukhtar A Sheikh Hospital, Multan, PAK; 3 Ear, Nose, and Throat, Combined Military Hospital, Multan, PAK; 4 Internal Medicine, ECMC Hospital, Buffalo, USA; 5 Internal Medicine, Khyber Medical College, Peshawar, PAK; 6 Internal Medicine, TidalHealth Penninsula Regional, Salisbury, USA; 7 Internal Medicine, Saidu Medical College, Saidu Sharif, PAK; 8 Psychology, National University of Modern Languages, Multan Campus, Multan, PAK; 9 Internal Medicine, Jinnah Hospital, Lahore, PAK; 10 Anthropology, Institute of Social and Cultural Studies, Multan, PAK

**Keywords:** gastrointestinal symptoms, gut-brain axis, migraine disability, vertigo, vestibular migraine

## Abstract

Background: Vestibular migraine (VM) is a condition characterized by regular attacks of vertigo and imbalance, along with migraine symptoms or signs. However, the role of the gut-brain axis (GBA) in mediating the severity of VM symptoms, particularly in the Pakistani population, remains poorly understood. This study aims to elucidate the relationship between GBA dysfunction and the severity of VM.

Methodology: This cross-sectional study was conducted from May 2024 to April 2025 at Jinnah Hospital, Lahore, in collaboration with the Neurology and ENT departments. Convenience sampling was employed to recruit 386 participants diagnosed with VM. Data were collected using three primary instruments: the Gastrointestinal Symptom Rating Scale (GSRS), the Migraine Disability Assessment Test (MIDAS), and the Vertigo Symptom Scale-Short Form (VSS-SF). Demographic and lifestyle data were also collected. Pearson correlation, independent t-tests, analysis of variance (ANOVA), and multiple linear regression were used to study the correlation between GBA dysfunction and VM severity.

Results: The sample included 386 participants, with males (296, 77%) and participants aged 60 years or older (154, 40%). Positive correlations were significant between gastrointestinal symptoms, migraine-related disability, and the severity of vertigo (*P* < 0.001; 95% confidence interval (CI) 0.06-0.18). Multiple linear regression analysis revealed that the severity of gastrointestinal symptoms (95% CI 0.15-0.55), older age (95% CI 2.70-7.70), and a high rate of vertigo incidences (95% CI 0.27-2.23) were significant predictors of increased migraine disability and vertigo symptom severity (*P* < 0.05). Anxiety and depression were also found to be related to severe symptoms, and this may indicate that mental health is also linked to GBA dysfunction in VM.

Conclusions: The study demonstrates an association between GBA-related symptoms and greater VM severity. These findings highlight the potential importance of assessing gastrointestinal and psychological comorbidities in the management of VM, particularly in Pakistani clinical settings where such factors may be overlooked. Future research should investigate the therapeutic potential of gut-targeted interventions using longitudinal designs and objective measures to clarify causal relationships and assess efficacy.

## Introduction

Vestibular migraine (VM) is a relatively unknown disorder that is manifested by periods of vertigo, lightheadedness, and loss of balance alongside migraine. Although earlier reports estimated its prevalence at up to 1%, more recent studies suggest a range of 1%-3% in the general population, with higher rates reported among patients with migraine (10% in Asia and up to 21% in Europe) [1,2]. These episodes last between five minutes and three days and may not always be accompanied by a headache; thus, diagnosis depends on related characteristics of a migraine and rules out other vestibular causes [3].

The diagnostic criteria for VM, developed by the Bárány Society and the International Headache Society (IHS), emphasize a history of migraine, recurrent vestibular symptoms, and both the completeness and severity of symptoms [4]. VM is now recognized as a distinct clinical entity, but its pathophysiology remains poorly understood. It is believed to affect central vestibular pathways and is now treated with general migraine treatment strategies [5]. Poor sleep quality and morning symptom onset are two lifestyle-modifiable factors that have been associated with the severity of VM [6]. Recent findings also suggest that diet, intestinal microbiota composition, and probiotics may potentially impact the frequency and severity of migraines, indicating the presence of a gut-brain axis (GBA) in VM pathogenesis [7].

Recent evidence has strengthened the link between the GBA and migraine. Specific bacterial taxa, such as Bifidobacteriaceae, have been associated with migraine subtypes. Systematic reviews have reported a reduction in Faecalibacterium and an increase in Veillonella among patients with migraine, suggesting that gut microbiota dysbiosis may contribute to migraine pathophysiology [8,9].

Migraine is usually comorbid with other gastrointestinal (GI) diseases, including gastroparesis, functional dyspepsia, and cyclic vomiting syndrome, which could determine the efficiency of treatment and clinical outcomes in general. Common pathophysiological pathways and gastroparesis implicate the need for targeted, non-oral treatments in migraine management [10]. Additionally, anxiety is frequently comorbid with vestibular deficits, as there may be overlapping neurological pathways and activity of the GBA. Any modification in the intestinal microbiome and the associated metabolites can be a possible cause of this comorbidity [11].

Based on this evidence, it is crucial to investigate the relationship between GBA dysfunction and the severity of VMs, which may reveal new therapeutic targets and enhance patient outcomes.

This study aims to investigate whether GBA dysfunction is associated with greater severity of VM symptoms in a Pakistani clinical population. Secondary objectives include examining GI symptom patterns, their association with migraine frequency and severity, and the influence of demographic and lifestyle factors on these patterns.

Rationale

VM is a severe illness that impacts the life of patients markedly, but the specific mechanisms of the development of the disease are unclear. Conventional research has primarily focused on neurological mechanisms, overlooking the potential role that systemic factors, such as gut health, may play in the development of these conditions. More recently, the GBA has been an area of growing interest, as it represents a complex exchange between GI mechanisms and brain function. Considering the concurring symptoms and similar pathological mechanisms between migraine and GI issues, impairment in this axis might affect the frequencies and levels of VM attacks.

In Pakistan, there is a lack of perception and identification of VM, and GI disturbances are not well noted or incorporated in neurological examinations. Moreover, there are significant differences in dietary habits, stressful lifestyles, and access to medical care across regions, which can contribute to the health situation of the gut and migraine. Investigating the relationship between GBA dysregulation and the severity of VM in this cohort may give an essential insight into culturally and geographically specific risk factors. The proposed research aims to address a substantial gap in the literature by contributing local evidence to an emerging international debate and providing a more holistic, context-specific management model for migraine treatment in Pakistan.

Study objectives and hypotheses

Primary Hypothesis

GBA dysfunction is significantly associated with greater severity of VM symptoms in a Pakistani clinical population.

Secondary Hypothesis

Patients with VM experience a higher occurrence of GI symptoms compared to those without VM.

Specific gut-related symptoms (e.g., bloating, abnormal bowel habits, food sensitivities) are positively associated with increased frequency and severity of VM attacks.

Demographic and lifestyle characteristics (including diet, stress, and medical comorbidities) significantly modify the relationship between GI-brain axis dysfunction and the degree of VM.

## Materials and methods

Research design and methods

This was a cross-sectional study. Patients were recruited consecutively over one year, but each participant was assessed only once at the time of enrollment. Individuals were recruited through the outpatient clinics of neurology and the ENT department of Jinnah Hospital, Lahore, which may not fully represent rural or underrepresented populations.

The information was collected using a structured questionnaire to evaluate the severity of VM and identify indicators of GBA impairment. As the questionnaire was only available in English, some participants required assistance from the research team to understand particular items, which was provided to minimize comprehension difficulties. Patients were approached by trained research assistants during routine clinic visits. Consent to participate in the study was obtained after an explanation of the study's purpose and details, along with sufficient time for discussion of the included questions. The study only involved individuals who signed a written or oral informed consent form. The respondents completed the questionnaire either independently, based on their level of literacy, or with the assistance of a research team member during the interviews. This approach ensured a respectful communication and accurate depiction of reality in the Pakistani healthcare environment, particularly in terms of patients' everyday lives.

Sampling strategy and population size

The target population was assumed to be infinite, as the precise number of adults with VM in Pakistan was unknown. The calculation of the sample size was done using the following formula:

\[n = \frac{Z^2 \cdot p (1 - p)}{d^2}\]

where *Z* refers to the *z*-score at the confidence level selected, *p* is the estimated prevalence of the characteristic to be located, and *d* is the margin of error acceptable. In this research, the level of confidence was 95%, corresponding to a *z*-score of 1.96, and the margin of error (*d*) was 0.05. A prevalence estimate (*p*) of 0.50 was chosen because it resulted in the maximum sample size possible, and it accounts for the variability in the population. A conservative prevalence estimate (*p* = 0.50) was selected in the absence of reliable local data, yielding a theoretical minimum sample size of 384 [12].

In practice, convenience sampling was used to recruit adult participants with symptoms of VM who were referred to the outpatient departments of Jinnah Hospital, Lahore, specifically in the neurology and ENT departments. During routine outpatient clinic visits, 415 individuals were approached. Trained research assistants screened all patients, and a neurologist or ENT specialist confirmed the diagnosis of VM according to the Bárány Society/International Headache Society (IHS) criteria [4]. Diagnosis required at least five lifetime vertigo episodes lasting between five minutes and 72 hours, with a history of migraine, and other vestibular disorders (e.g., Meniere's disease, vestibular neuritis) were ruled out. Among them, 386 participants joined the study and completed the questionnaires, indicating a response rate of approximately 93%. Twenty-nine respondents were excluded from the final analysis due to non-participation, withdrawal during data collection, or incomplete responses. Participants who had fully analyzable and usable data were selected for the study. This approach ensured that, despite using convenience sampling, the achieved sample size exceeded the theoretical requirement, thereby maintaining sufficient study power.

Inclusion criteria

The study participants were adults (≥18 years) with VM, diagnosed by a qualified neurologist or ENT specialist according to Bárány/IHS criteria. A diagnosis required at least five lifetime episodes of vertigo lasting five minutes to 72 hours, with a history of migraine and a temporal association between vestibular and migraine symptoms. Only individuals who could comprehend the study process, provide written informed consent, and complete the questionnaire with or without assistance were regarded as eligible. Including both male and female patients from diverse socioeconomic backgrounds was intended to ensure a more representative sample.

Exclusion criteria

Participants who presented a specific history of other central or periphery vestibular conditions (including vestibular neuritis or Meniere disease) or presented mental health conditions of serious degree that might obstruct their capability to understand the questionnaire were not included. Patients with severe GI disorders, regardless of GBA impairment (e.g., inflammatory bowel disease or GI malignancies), were also excluded. Additionally, participants with incomplete responses or those who dropped out during data collection were excluded from the final analysis; no imputation techniques were applied, and only fully completed questionnaires were analyzed.

Data collection tools

To collect relevant and comprehensive data within the framework of this study, a structured questionnaire was created, comprising four primary parts: demographic details, vertigo-related symptoms, migraine-related disability, and GI symptomatology. The questionnaire aimed to test the correlation between the GBA disturbance and the intensity of the VM symptomatology in the clinical population of the Pakistani population. The original English version of all the standardized instruments was used because it was in the English style, allowing participants to understand and respond to the items. The scales were not linguistically and culturally adapted.

Demographic information

The initial section of the questionnaire collected demographic and clinical background data to establish potential relationships with the severity of VM. This ranged from simple information, such as age, gender, marital status, level of education, and job status. Moreover, it contained screening questions addressing whether participants experienced periodic vertigo or dizziness attacks, had any GI problems (e.g., bloating, constipation, reflux), had any psychological illnesses (e.g., anxiety or depression), or were taking any migraine or GI medications. They served to present a broader clinical context of the participants and identify possible comorbidities associated with GBA dysregulation (Appendix). It should be noted that the assessment of psychological illnesses was based on self-report rather than validated instruments (e.g., Patient Health Questionnaire-9 (PHQ-9), Generalized Anxiety Disorder-7 (GAD-7)), which may limit the reliability of these findings.

Vertigo Symptom Scale-Short Form (VSS-SF)

The second part included the VSS-SF, a valid self-report questionnaire developed by Yardley et al.[13]. It measures the severity and frequency of vertigo and other autonomic symptoms during the previous month. VSS-SF consists of 15 questions, divided into two subscales: vestibular-balance symptoms (eight questions) and autonomic-anxiety symptoms (seven questions). All items are graded on a 5-point Likert scale, with the lowest response (*never*) assigned a score of 0 and the highest response (*very often*) assigned a score of 4. This results in a cumulative point system for the symptom scale ranging from 0 to 60, with higher scores indicating more intense symptoms. The internal consistency of the tool is high, with Cronbach's alpha coefficients ranging from 0.87 to 0.90, indicating excellent reliability. The VSS-SF is a widely used tool in clinical practice and research, where it can be employed to measure the burden of vestibular symptoms and track their progression over time. Cultural and linguistic adjustments were not made to maintain the validity and reliability of the instrument [13].

Migraine Disability Assessment Test (MIDAS) questionnaire

The third part consisted of the MIDAS questionnaire, a documented clinical instrument developed and authored by Dr. Richard B. Lipton and associates in 2000, to assess the functional effects of migraine on a person's daily activities. The MIDAS questionnaire includes five key questions that require the respondent to indicate the number of days within the last three months that migraine has impacted normal functioning, such as employment, schooling, home responsibilities, and social or leisure activities. There are also two unscored questions regarding how often one experiences headaches and whether they hurt. The five scored items are answered using a numerical value, which is the number of days affected. These five responses are summed to obtain the total MIDAS score, with higher scores indicating higher levels of disability caused by migraine. The scores are typically categorized into four disability levels, including Grade I (0-5 days with little or no disability), Grade II (6-10 days, mild), Grade III (11-20 days, moderate), and Grade IV (21+ days, severe). The questionnaire has been demonstrated to be relatively reliable in both clinical and community settings, with an average Cronbach's alpha of 0.78 to 0.83, indicating good internal consistency. The MIDAS questionnaire used in this study was the original English version and had not been translated or altered in any way [14].

Gastrointestinal Symptom Rating Scale (GSRS)

The last part of the questionnaire was the GSRS, a standardized self-administered assessment of the prevalence and intensity of GI symptoms created by Svedlund et al. in 1988 [15]. The GSRS consists of 15 items categorized into five clinically significant scales: reflux, abdominal pain, indigestion, diarrhea, and constipation. All items are measured on a 7-point Likert scale, where 1 indicates no discomfort at all and 7 indicates very severe pain, with higher scores indicating more significant GI symptoms. The overall GSRS total is typically computed as the mean of item scores at either the global or domain level, depending on the emphasis of the analysis. The internal consistency of the scale has been demonstrated to be fair, yielding Cronbach's alpha values ranging from 0.74 to 0.85 across different domains in clinical groups. It is applied very broadly in both research and clinical contexts to assess the burden of GI symptoms, particularly concerning gut-brain interaction disorders. In this research, the translation and cultural adaptation of the GSRS were not performed; the original English version was used [15].

Permissions for questionnaires

The VSS-SF is distributed under a Creative Commons license, permitting its use in non-commercial academic research without additional permission, provided appropriate citation is given. Permission to use the MIDAS questionnaire was obtained from Mapi Research Trust (Special Terms No. 120209), and permission to use the GSRS was granted by AstraZeneca AB (License No. 556011-7482).

Procedure

The recruitment of participants took place in the neurology and ENT outpatient departments of Jinnah Hospital, Lahore, following a signed agreement of informed consent. Data were collected from May 2024 to April 2025. The eligibility criteria were assessed as the participants attended their routine outpatient visits, and the study purpose was discussed in their local language (Urdu or English) according to their choice. Questionnaires were administered either self-completed or with standardized assistance from research staff, depending on their ability to read and write, and their comfort with answering the questions independently. Assistance involved reading the questions aloud neutrally, without leading responses, to ensure consistent administration across participants. Average completion time was approximately 20-30 minutes per participant. All data were treated anonymously, and no personally identifiable information was collected, ensuring the confidentiality of participants and adherence to ethical standards. This method enabled culturally responsive and respectful communication with participants from diverse socioeconomic and educational backgrounds, facilitating efficient and contextually effective data gathering.

Analytical approach

IBM SPSS Statistics version 26 (IBM Corp., Armonk, NY) was used to process and analyze data. Demographic variables were summarized using frequencies and percentages as descriptive statistics, including age, gender, marital status, educational level, occupation, and results of vertigo, GI issues, and psychological condition. The Kolmogorov-Smirnov and Shapiro-Wilk statistics were used to define the normality of the distribution of the GSRS, MIDAS, and VSS - SF. Pearson correlation analysis was employed to identify the correlations between the scores of GSRS, MIDAS, and VSS - SF. A comparison of the mean scores of participants with and without psychological conditions on these scales was conducted using independent samples t-tests. One-way ANOVA was used to detect differences in GSRS, MIDAS, and VSS - SF between age groups and vertigo frequency groups.

Additionally, a multiple linear regression analysis was used to establish predictors of MIDAS and VSS - SF scores, including GI symptoms, age, gender, occurrence of vertigo, and GI problems. Finally, a chi-square test was used to evaluate the relationships between the categorical variables, including vertigo frequency and GI issues, as well as vertigo frequency and current medication use. A significance level of *P* < 0.05 was used to interpret all the statistical tests.

Ethical protocols

The study was conducted ethically and adhered to the standard ethical principles of research that govern the use of human subjects. The Institutional Review Board (IRB) of Jinnah Hospital, Lahore (Approval No. 161/02/05/2024/S2 ERB), reviewed and approved the study protocol. The study was conducted in accordance with core ethical principles, including respect for persons, beneficence, and confidentiality. Each participant was thoroughly informed of the aims, process, and possible risks and benefits of the research before participating. Each participant provided written or verbal informed consent before data collection began. Participation in the study was entirely voluntary, and participants were informed that they could withdraw at any time without adverse consequences. Anonymity was ensured by removing personal identifiers and maintaining the confidentiality of all data. The research team also ensured the anonymity of participants throughout the data handling and analysis process. Questionnaires were assessed for completeness, and where critical responses were lacking, especially in cases where standardized instruments like VSS-SF, MIDAS, or GSRS were not complete, such questionnaires were excluded from the final usable dataset to maintain the validity and integrity of the findings.

## Results

Table 1 presents the demographic and clinical characteristics of the sample (*N* = 386), of whom 154 (40%) were 60 years or older, making this the largest age category. Most of them were male (*N* = 296, 77%) and married (*N* = 142, 37%), with a significant number also being divorced (*N* = 106, 27%). It should be noted that the male predominance and relatively high divorce rate are atypical for VM cohorts and may reflect the recruitment setting and convenience sampling approach rather than the actual population distribution. Education-wise, 106 (27%) were only at the primary level of education, whereas fewer had a bachelor's degree (*N *= 22, 6%) or a master's degree (*N* = 8, 2%). Occupational status revealed that the number of people employed (*N* = 136, 35%) was higher than that of unemployed people (*N* = 108, 28%). As far as symptoms were concerned, 59 (15%) had frequent vertigo or dizziness, and 143 (37%) had frequent gastrointestinal problems. Many participants (*N* = 230, 60%) said that they were diagnosed with a psychological condition like anxiety or depression. Regarding treatment, 165 (43%) received medication for GI problems, 137 (35%) received medical treatment for migraines, and 67 (17%) received medication for both problems.

**Table 1:** Demographic characteristic of participants (N = 386). *f*, frequency Values are presented as *N* (%), *N* = 386. No statistical comparisons were performed for demographic variables in this table.

Variable	f	%
Age (years)		
18-25	28	7
26-35	48	12
36-45	60	15
46-59	96	25
60 and above	154	40
Gender		
Male	296	77
Female	90	23
Marital status		
Single	100	26
Married	142	37
Divorced	106	27
Widowed	38	10
Educational level		
No formal education	82	21
Primary	106	27
Matric/o-level	97	25
Intermediate/A-level	71	18
Bachelor's degree	22	6
Master's degree	8	2
Occupation		
Student	70	18
Employed	136	35
Unemployed	108	28
Retired	72	19
Do you experience vertigo or dizziness episodes regularly?		
Yes, frequently	59	15
Occasionally	130	34
Rarely	134	35
Never	63	16
Do you have any gastrointestinal (GI) issues (e.g., bloating, constipation, reflux)?		
Yes, frequently	143	37
Occasionally	138	36
Rarely	77	20
Never	28	7
Have you been diagnosed with any psychological condition (e.g., anxiety, depression)?		
Yes	230	60
No	156	40
Do you currently use any medication for migraine or GI issues?		
Yes - for migraine	137	35
Yes - for GI issues	165	43
Yes - for both	67	17
No medication	17	4

Table 2 presents the data on the evaluation of normality for the GSRS, MIDAS, and VSS - SF among the participants (*N* = 386), using both the Kolmogorov-Smirnov and Shapiro-Wilk approaches to measure normality. The Kolmogorov-Smirnov test for the GSRS yielded a significant *P*-value (*P* = 0.012), indicating that the GSRS was not normally distributed; however, the Shapiro-Wilk test (*P* = 0.339) was not significant, suggesting that the data can still be considered normally distributed. The results for both the MIDAS and VSS - SF were not significant (Kolmogorov-Smirnov, *P* = 0.200; Shapiro-Wilk, *P* = 0.084 and 0.122, respectively), supporting the assumption of normality. Based on these findings, all three variables can be considered normally distributed, and the application of parametric statistical tests is therefore appropriate.

**Table 2:** Tests of normality for the Gastrointestinal Symptom Rating Scale, Migraine Disability Assessment Test, and Vertigo Symptom Scale-Short Form. Values are presented as test statistics (df = 386) with corresponding *P*-values. A *P*-value < 0.05 was considered statistically significant, indicating deviation from normal distribution (*N* = 386). df, degree of freedom

Variables	Kolmogorov-Smirnov			Shapiro-Wilk		
	Statistic	df	P	Statistic	df	P
Gastrointestinal Symptom Rating Scale	0.053	386	0.012	0.996	386	0.339
Migraine Disability Assessment Test	0.041	386	0.200	0.991	386	0.084
Vertigo Symptom Scale-Short Form	0.037	386	0.200	0.989	386	0.122

Table 3 presents the Pearson correlation coefficients of the GSRS, MIDAS, and VSS-SF in the participant sample (*N* = 386). The GSRS showed a significant positive correlation with the MIDAS (*r* = 0.22, *P* < 0.001) and the VSS - SF (*r* = 0.20, *P* < 0.001). On the same note, the MIDAS showed a positive correlation with the VSS -SF, with a significant correlation (*r* = 0.18, *P* = 0.001). These findings indicate that increasing levels of GI symptoms correlate with rising levels of migraine-related disability and vertigo symptoms and that migraine disability is moderately related to the level of vertigo. Each of the correlations was significant at the 0.01 level, indicating a strong correlation.

**Table 3:** Pearson correlation between the Gastrointestinal Symptom Rating Scale, Migraine Disability Assessment Test, and Vertigo Symptom Scale-Short Form (N = 386). Values represent Pearson correlation coefficients (*r*) between continuous variables, *N* = 386; *P* < 0.01 (two-tailed) was considered statistically significant and is denoted by double asterisks (**).

Variable	1	2	3	P
Gastrointestinal Symptom Rating Scale	-	0.22^**^	0.20^**^	<0.001^**^
Migraine Disability Assessment Test	-	-	0.18^**^	0.001^**^
Vertigo Symptom Scale-Short Form	-	-	-	0.001^**^

Table 4 presents the findings of independent samples t-tests comparing the severity of symptoms between individuals with psychological conditions (*N* = 230) and those without (*N* = 156). The patients with psychological diagnoses demonstrated significantly greater scores on the GSRS (*M* = 50.28, standard deviation (SD) = 6.90) than those with a negative result (*M* = 47.20, SD = 6.00), *t*(384) = 3.950, *P* < 0.001, with a moderate effect size (Cohen's *d* = 0.47). Similarly, the MIDAS scores were higher among respondents with psychological diagnoses (*M* = 21.10, SD = 13.00) compared to those without (*M* = 16.50, SD = 11.20), *t*(384) = 3.37, *P* = 0.001, *d* = 0.37. Respondents with psychological conditions had higher scores on the VSS - SF (*M* = 42.90, SD = 5.20) compared to those without (*M* = 40.60, SD = 4.50), *t*(384) = 4.48, *P* < 0.001, *d* = 0.47. These results indicate that psychological conditions are strongly associated with greater symptom severity in GI, migraine, and vertigo domains.

**Table 4:** Independent samples t-tests for Gastrointestinal Symptom Rating Scale, Migraine Disability Assessment Test, and Vertigo Symptoms Scale - Short Form by psychological conditions. Values are presented as mean ± standard deviation. Independent samples t-tests were conducted to compare participants with and without a psychological diagnosis. Group sizes are shown as *N* (%). Reported statistics include *P*-values, *t*-values, 95% confidence intervals (CIs), and effect sizes (Cohen’s *d*). A *P*-value < 0.05 was considered statistically significant (*N* = 386).

Variable	Yes (*N *= 230, 60%) (M ± SD)	No (*N *= 156, 40%) (M ± SD)	t	P	95% CI LL	95% CI UL	Cohen’s *d*
Gastrointestinal Symptom Rating Scale	50.28 ± 6.90	47.20 ± 6.00	3.950	<0.001^**^	1.55	4.61	0.47
Migraine Disability Assessment Test	21.10 ± 13.00	16.50 ± 11.20	3.374	0.001^**^	1.92	7.28	0.37
Vertigo Symptoms Scale - Short Form	42.90 ± 5.20	40.60 ± 4.50	4.481	<0.001^**^	1.29	3.31	0.47

Table 5 shows that symptom severity in the GI, migraine, and vertigo dimensions increased significantly with age. One-way ANOVA results indicated that older adults scored higher on all three measures, with statistically significant differences between age groups (all *P* < 0.001). Vertigo symptoms showed the largest age-related effect (*η*² = 0.163), followed by migraine disability (*η*² = 0.067) and GI symptoms (*η*² = 0.045). These findings indicated a significant increase in the severity of the symptoms with age, especially the condition of vertigo.

**Table 5:** One-way analysis of variance (ANOVA) comparing Gastrointestinal Symptom Rating Scale, Migraine Disability Assessment Test, and Vertigo Symptoms Scale-Short Form by age group (N = 386). Data are presented as mean ± standard deviation (M ± SD). Group sizes are shown as *N* (%). One-way ANOVA was conducted to examine the effect of age on symptom scores. A *P*-value < 0.05 was considered statistically significant. *η*² represents partial eta-squared effect size.

Variable	18-25 years (*N *= 28, 7%) (M ± SD)	26-35 years (*N *= 48, 12%) (M ± SD)	36-45 years (*N *= 60, 15%) (M ± SD)	46-59 years (*N *= 96, 25%) (M ± SD)	60 years and above (*N *= 154, 40%) (M ± SD)	P	*F*(4,381)	*η*^2^
Gastrointestinal Symptom Rating Scale	47.10 ± 6.50	48.80 ± 6.10	50.20 ± 5.80	51.60 ± 5.00	53.00 ± 4.90	<0.001**	8.93	0.045
Migraine Disability Assessment Test	17.00 ± 11.00	21.50 ± 13.00	26.00 ± 13.50	30.00 ± 13.20	35.00 ± 13.00	<0.001**	13.81	0.067
Vertigo Symptom Scale-Short Form	41.20 ± 4.30	42.80 ± 4.60	44.10 ± 4.80	45.80 ± 4.50	47.50 ± 3.80	<0.001**	37.31	0.163

Table 6 presents the results of a one-way ANOVA comparing symptom severity across four groups, based on the self-reported frequency of vertigo episodes (frequently, occasionally, rarely, and never) among participants (*N* = 386). All three variables showed significant differences. On the GSRS, there was a progressive decrease in the mean scores of subjects experiencing vertigo more frequently (*M* = 51.20 ± 6.50), those with less frequent vertigo (*M *= 50.50 ± 6.67), those who experienced it occasionally (*M *= 48.60 ± 7.03), and those who never had vertigo (*M *= 45.40 ± 5.90), *F*(3,382) = 7.02, *P *< 0.001. A similar pattern was observed for the MIDAS, with the mean score in the frequent group being higher (*M* = 24.30 ± 46.00) and significantly lower in the never group (*M *= 14.50 ± 8.40), *F*(3,382) = 5.88, *P *= 0.001, *η*^2^ = 0.044. The most substantial effect was observed on the VSS - SF, with mean values of 44.60 ± 4.30 (frequent), 39.40 ± 4.30 (much less), 37.10 ± 4.20 (somewhat less), and 39.50 ± 4.20 (never), *F*(3, 382) = 20.75, *P* < 0.001, *η*² = 0.140. These findings suggest that individuals with vertigo tend to report significantly higher levels of gastrointestinal symptoms, migraine-related disability, and vertigo severity, with small to moderate effect sizes.

**Table 6:** One-way analysis of variance (ANOVA) comparing the Gastrointestinal Symptom Rating Scale, Migraine Disability Assessment Test, and Vertigo Symptom Scale-Short Form by frequency of vertigo episodes (N = 386). Data are presented as mean ± standard deviation (M ± SD). Group sizes are reported as *N* (%). One-way ANOVA was used to compare symptom scores across frequency categories. A *P*-value < 0.05 was considered statistically significant, and η² represents the partial eta-squared effect size.

Variable	Yes, frequently (*N *= 59, 15%) (M ± SD)	Occasionally (*N *= 130, 34%) (M ± SD)	Rarely (*N *= 134, 35%) (M ± SD)	Never (*N *= 63, 16%) (M ± SD)	P	*F*(3,382)	*η*^2^
Gastrointestinal Symptom Rating Scale	51.20 ± 6.50	48.80 ± 6.70	47.10 ± 6.40	45.40 ± 5.90	<0.001^**^	7.02	0.052
Migraine Disability Assessment Test	24.30 ± 16.00	20.00 ± 13.00	16.90 ± 11.20	14.50 ± 8.40	0.001^**^	5.88	0.044
Vertigo Symptom Scale-Short Form	44.60 ± 4.30	42.70 ± 4.80	40.80 ± 4.60	39.50 ± 4.20	<0.001^**^	20.75	0.140

Table 7 presents the findings of two multiple linear regression models, which include the migraine disability score and vertigo symptom scores as outcomes. In the case of the MIDAS, it was significantly predicted that people with higher GI symptom burden (*B* = 0.35, *P* = 0.001), older age (*B* = 5.20, *P* < 0.001), female gender (*B* = 2.85, *P* = 0.018), more frequent vertigo episodes (*B* = 1.25, *P* = 0.013), and GI problems would have higher scores. All these predictors suggest that migraine-related disability is influenced not only by physical symptoms but also by demographic factors. In the VSS - SF, higher scores were significantly associated with GI symptoms (*B* = 0.12, *P* < 0.001), older age (*B* = 1.95, *P* = 0.002), female gender (*B* = 1.35, *P* = 0.008), more frequent vertigo attacks (*B* = 1.10, *P* < 0.001), and GI problems (*B* = 0.95, *P* = 0.001). These findings reiterate the comorbidity of GI complaints, age, and gender with the severity of vertigo symptoms, supporting the possible involvement of gut-brain and emotional-vestibular connections in symptom development.

**Table 7:** Multiple linear regression analysis predicting Migraine Disability Assessment Test and Vertigo Symptom Scale-Short Form scores (N = 386). Multiple linear regression was conducted to identify predictors of migraine disability and vertigo symptoms. Values reported include unstandardized coefficients (*B*), 95% confidence intervals (CIs), standard errors (SEs), standardized beta coefficients (β), and *P*-values. A *P*-value < 0.05 was considered statistically significant (*N* = 386).

Outcome Variable	Predictor	B	95% CI LL	95% CI UL	SE	β	P
Migraine Disability Assessment Test	Constant	4.210	-5.823	14.243	5.100	-	0.410
^-^	Gastrointestinal Symptom Rating Scale	0.35	0.15	0.55	0.10	0.17	0.001^**^
^-^	Age	5.20	2.70	7.70	1.25	0.17	<0.001^**^
^-^	Gender	2.85	0.49	5.21	1.20	0.11	0.018^**^
^-^	Vertigo episodes	1.25	0.27	2.23	0.50	0.10	0.013^**^
^-^	Gastrointestinal issues	1.60	0.42	2.78	0.60	0.12	0.008^**^
Vertigo Symptom Scale-Short Form	Constant	36.25	32.51	39.99	1.90	-	<0.001^**^
^-^	Gastrointestinal Symptom Rating Scale	0.12	0.06	0.18	0.03	0.18	<0.001^**^
^-^	Age	1.95	0.85	3.05	0.55	0.16	0.002^**^
^-^	Gender	1.35	0.37	2.33	0.50	0.12	0.008^**^
^-^	Vertigo episodes	1.10	0.58	1.62	0.26	0.21	<0.001^**^
^-^	Gastrointestinal issues	0.95	0.40	1.50	0.28	0.16	0.001^**^

**Figure 1:**
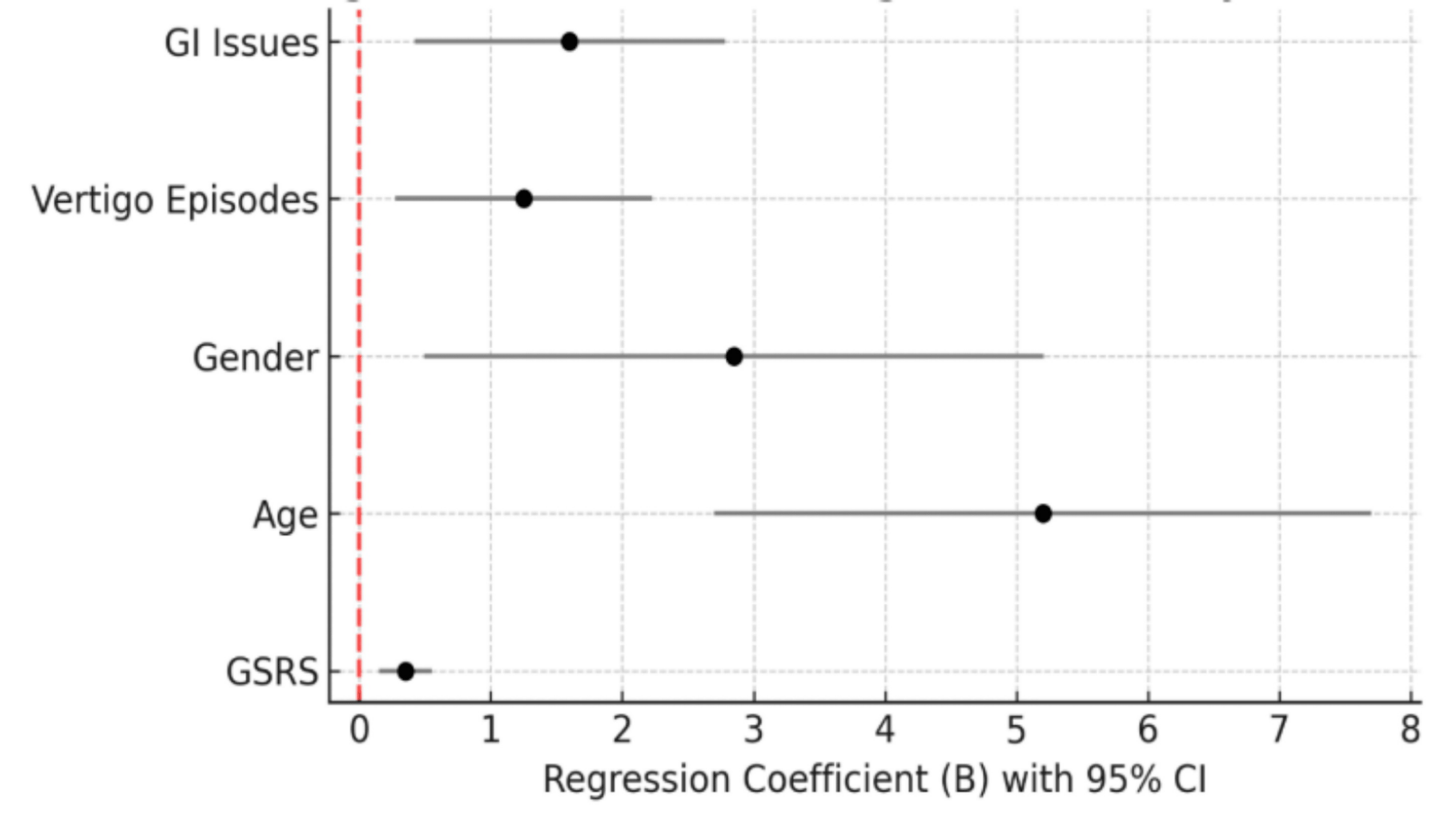
Regression coefficients and 95% confidence intervals for predictors of migraine disability (MIDAS). Significant predictors included gastrointestinal symptom severity (GSRS), age, gender, frequency of vertigo episodes, and gastrointestinal problems. MIDAS, Migraine Disability Assessment Test; GSRS, Gastrointestinal Symptom Rating Scale; CI, confidence interval; GI, gastrointestinal

Figure 1 presents regression coefficients (B) with 95% confidence intervals for predictors of migraine disability (MIDAS). The results indicate that gastrointestinal symptom severity (GSRS), age, gender, frequency of vertigo episodes, and GI complaints were key predictors. GSRS had the highest effect size, followed by age, which indicates that greater severity of GI symptoms and older age are strongly linked to greater migration disability. Gender and vertigo were other important factors, and GI problems were moderate contributors. Confidence intervals that do not cross zero confirm the statistical significance of these predictors, highlighting their role in explaining variability in migraine-related disability.

**Figure 2:**
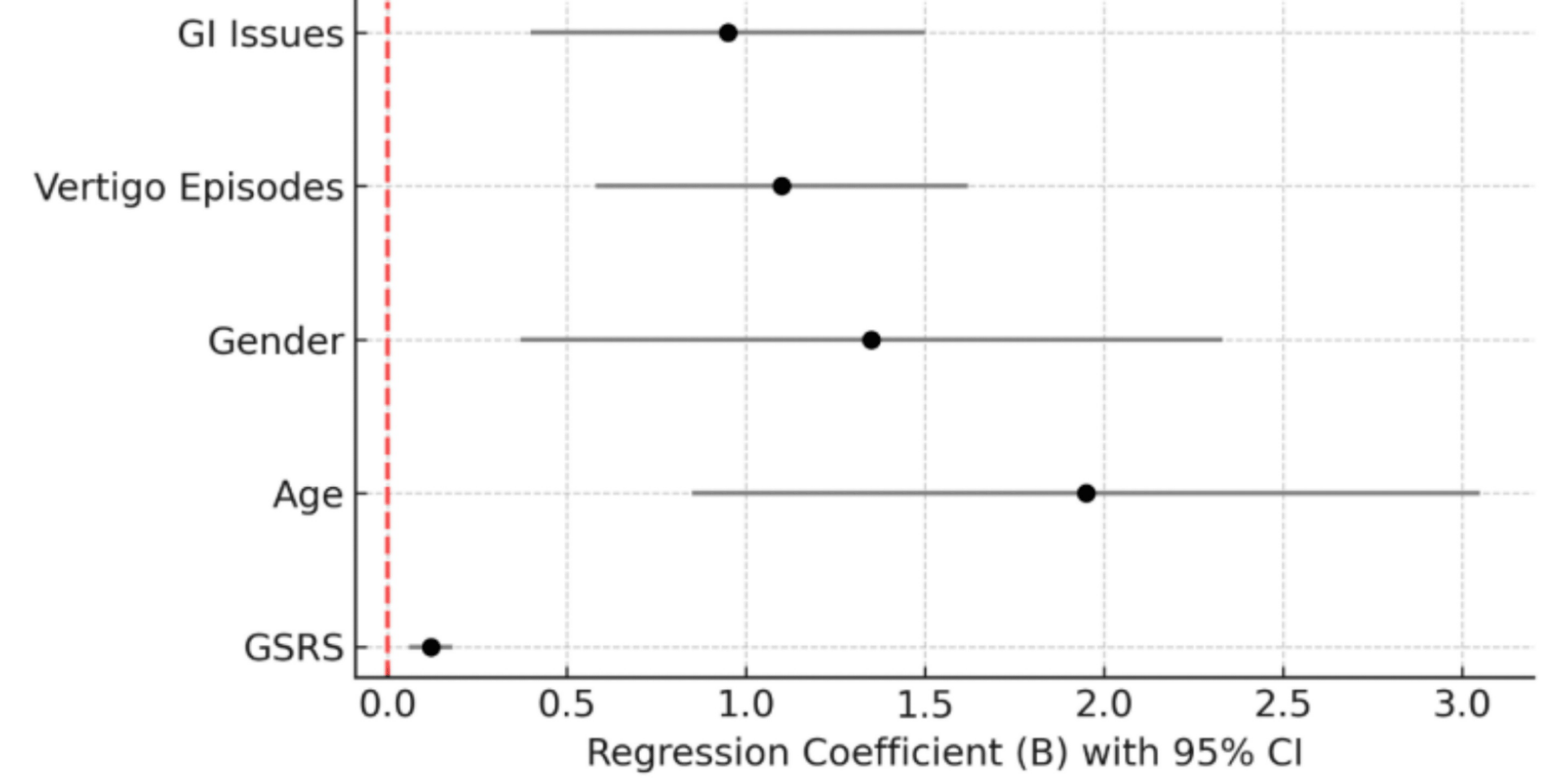
Regression coefficients and 95% confidence intervals for predictors of vertigo symptoms. Significant predictors included gastrointestinal symptom severity (GSRS), age, gender, frequency of vertigo episodes, and gastrointestinal problems GSRS, Gastrointestinal Symptom Rating Scale; CI, confidence interval; GI, gastrointestinal

Figure 2 depicts the 95% confidence intervals of the regression coefficients for predictors of vertigo symptoms. The analysis indicates that GI symptom severity (GSRS), GI problems, age, gender, and the frequency of vertigo episodes were all significant predictors. GSRS and age showed the strongest correlation with vertigo symptoms, with GSRS followed by the frequency of vertigo episodes. This indicates that individuals with more severe GI symptoms, older age, and frequent vertigo episodes are more likely to report higher vertigo symptom scores. Other contributing factors included gender and GI problems. Because the confidence intervals for these predictors do not cross zero, their effects are statistically significant, highlighting their importance in explaining variability in vertigo symptoms.

Table 8 shows the prevalence of vertigo/dizziness attacks in association with GI problems. The association between the two variables was statistically significant, as indicated by a Chi-square test (χ² = 24.8, *P* = 0.003), showing that individuals who experience gastrointestinal problems more frequently are more likely to report vertigo or dizziness. The data show that 59 participants (40%) who experience frequent vertigo also reported frequent GI problems. Also, 130 participants (48%) reported experiencing GI disorders accompanied by vertigo occasionally. As the frequency of vertigo decreases, its association with gastrointestinal problems becomes weaker. The *P*-value of 0.003 indicates that this relationship is unlikely to have occurred by chance.

**Table 8:** Frequency of vertigo/dizziness episodes by gastrointestinal issues. Data are presented as *N* (%). The Chi-square test was used to assess the relationship between vertigo/dizziness episodes and gastrointestinal problem frequency. Statistical significance was set at *P* < 0.05.

Vertigo/dizziness episodes	Yes, Frequently, *N* (%)	Occasionally, *N* (%)	Rarely, *N* (%)	Never, *N* (%)	Total	*χ*² (df = 9)	*P*-value
Yes, frequently	59 (40%)	35 (23%)	18 (12%)	5 (3%)	117	-	-
Occasionally	130 (48%)	48 (18%)	51 (19%)	24 (9%)	253	-	-
Rarely	134 (48%)	38 (14%)	47 (17%)	39 (14%)	258	-	-
Never	63 (35%)	12 (7%)	14 (8%)	28 (15%)	117	-	-
Total	-	-	-	-	-	24.8	0.003^**^

Table 9 examines the association between vertigo/dizziness attacks and the current migraine or GI medication usage. The Chi-square test results (χ² = 32.6, *P* < 0.001) indicate a strong association between the type of medications used and the incidence of vertigo. The results indicate that 25 patients (20%) with both migraine and GI issues experience frequent vertigo, and 50 patients (20%) with both conditions experience vertigo periodically. A similar pattern was observed across all groups, with individuals using migraine or GI medications most likely to report frequent or occasional vertigo. A *P*-value <0.001 indicates that this relationship is statistically significant, suggesting that current use of migraine or GI medication is associated with the prevalence of vertigo/dizziness episodes.

**Table 9:** Frequency of vertigo/dizziness episodes by current use of migraine or gastrointestinal (GI) medication. Data are presented as *N* (%). The Chi-square test was used to assess the association between vertigo/dizziness episodes and current use of migraine or GI medication. **Statistical significance was set at *P* < 0.001.

Vertigo/dizziness episodes	Yes - Migraine, *N* (%)	Yes - GI issues, *N* (%)	Yes - Both, *N* (%)	No medication, *N* (%)	Total	*χ*² (df = 9)	*P*-value
Yes, frequently	15 (12%)	10 (8%)	25 (20%)	9 (7%)	59	-	-
Occasionally	26 (10%)	35 (14%)	50 (20%)	19 (7%)	130	-	-
Rarely	31 (12%)	40 (15%)	45 (17%)	18 (7%)	134	-	-
Never	11 (9%)	9 (8%)	22 (19%)	21 (18%)	63	-	-
Total	-	-	-	-	-	32.6	<0.001^**^

## Discussion

This study explored the relationship between GBA dysfunction and the extent of VM symptoms in a clinical group of Pakistanis. In our research, GI symptoms were significantly associated with migraine-related disability, suggesting a potential role for GBA alterations and supporting previous literature on the GBA. These results highlight that GI comorbidities may influence migraine severity and should be considered when individualizing treatment plans [16]. Similarly, a small but significant correlation between GI symptoms and vertigo severity supports a possible gut-vestibular link, consistent with prior studies that have reported a notable connection between gastroesophageal reflux disease (GERD) and peripheral vertigo, which may be mediated by inflammation caused by reflux or the involvement of the middle ear [17]. Migraine-related disability was also associated with vertigo symptom severity, supporting previous findings in patients with Benign Paroxysmal Positional Vertigo (BPPV), where those with migraine experienced greater dizziness, imbalance, and impaired quality of life compared with patients without migraine [18].

The results of our study indicate that participants with psychological conditions were more likely to report GI symptoms, further supporting the association between mental well-being and GI discomfort. This aligns with previous research showing higher prevalence and greater severity of anxiety and depression in patients with Irritable Bowel Syndrome (IBS) and ulcerative colitis compared to healthy controls, suggesting a bidirectional gut-brain relationship [19]. Participants with psychological conditions reported higher migraine-related disability, consistent with studies showing that factors like pain catastrophizing and depressiveness significantly increase disability beyond migraine symptoms. These findings highlight the need to consider psychological comorbidities in migraine management [20]. We found that participants with psychological conditions experienced more vertigo symptoms, consistent with evidence from patients with BPPV, where anxiety and depression significantly mediated the relationship between sleep quality and vertigo severity. These findings highlight the importance of considering psychological factors in the management of vertigo [21].

Age was positively associated with GI symptoms, migraine disability, and vertigo severity. The oldest age group (≥60 years) reported the highest GSRS scores, consistent with physiological and pathological GI changes that accumulate with aging [22]. Migraine-related disability was also more pronounced among older adults, in line with prior studies showing that chronic migraine in later life is compounded by comorbidities and limited treatment options [23]. Similarly, vertigo-related disability increased with age, supporting previous findings that older individuals experience more severe dizziness and functional limitations, despite often reporting fewer classic symptoms [24].

In our research, participants who reported frequent vertigo also experienced a higher number of GI complaints, suggesting a potential link between vestibular and GI function. A prospective cohort study supports this association, showing that treatment of GI symptoms resulted in a significant reduction in dizziness, even when vertigo itself was not directly treated [25]. Additionally, a higher frequency of vertigo episodes was associated with greater migraine-related disability, indicating that vestibular symptoms contribute meaningfully to patients' daily disability. This observation aligns with prior research showing that migraine patients, particularly those with aura or chronic migraine, experience more severe vestibular handicaps and associated functional limitations [26]. Finally, vertigo severity was strongly correlated with episode frequency, consistent with earlier studies that have demonstrated that individuals with more frequent vestibular episodes report greater disruption in daily activities and reduced quality of life compared to those with less frequent or no vestibular dizziness [27].

Greater severity of GI symptoms was associated with higher migraine-related disability, consistent with evidence supporting a link between GBA dysfunction and migraine severity [15]. Older age and female sex were also associated with higher disability, reflecting known age-related vulnerability and hormonal influences in migraine [23,28]. Frequent vertigo attacks correlated with increased migraine-related disability, highlighting the contribution of vestibular symptoms to overall burden [26]. GI problems were particularly linked to migraine-related disability, in line with reports that functional GI disorders are often accompanied by higher anxiety, depression, and disability [29].

GI symptom severity was also associated with vertigo severity, supporting potential gut-brain interactions. Older participants and females reported more severe vertigo, consistent with age-related vestibular decline and female predisposition to vertigo, dizziness, and unsteadiness [24,30]. Finally, GI problems were linked to higher vertigo scores, consistent with evidence that autonomic dysfunction can contribute to dizziness in GI disorders [25,31].

Our research showed a substantial relationship between vertigo and GI conditions, indicating the role of the GBA in VM. Similarly, another paper revealed that the purely GI treatment of symptoms caused a significant decrease in dizziness, which corroborates the relation [25]. Participants with more frequent vertigo were also more likely to use medications for migraine or GI disorders, aligning with literature emphasizing tailored pharmacological treatment of vertigo based on underlying causes, such as migraine or intestinal disorders [32].

The findings support the notion that VM severity is associated with demographic, psychological, and GI-related factors, highlighting the multifactorial nature of the condition. While these relationships suggest potential directions for future research, they do not necessarily imply causality.

The results are especially applicable in the Pakistani setting, as VM is underdiagnosed in the region and GI symptoms are often overlooked in neurology clinics. The research emphasizes the significance of a multidisciplinary, integrative approach to assessing and treating migraine, involving screening for GI and psychological symptoms to enhance treatment outcomes.

Limitations

This research has certain limitations. First, the cross-sectional design does not allow for clear conclusions regarding whether one condition (GBA dysfunction) causes another (VM severity). Second, using self-reported questionnaires has another drawback, as it opens the possibility of response bias, especially among individuals with low health literacy. Third, convenience sampling was used in the recruitment of the sample, which was drawn from specific hospitals within an urban setting and may not accurately reflect the general population of the country, particularly in rural or underrepresented areas. Additionally, the generalizability of the findings to the entire VM population may be hindered by the sample composition with a male bias, as well as a larger-than-average number of people being divorced. Fourth, because the questionnaire was only available in English, some participants required assistance from the research team to understand particular items. While this support reduced comprehension errors, it may have introduced interviewer bias nonetheless. The lack of a formally translated and culturally validated version of the questionnaire remains a limitation. Fifth, the psychological conditions were evaluated based solely on self-report, and no clinical diagnostic instruments (e.g., PHQ-9, GAD-7) were used to validate these mental health variables. Sixth, there were possible confounding conditions, such as diet, medications consumed, comorbidities (diabetes and high blood pressure), and socioeconomic factors, that were not adjusted for in the regression models, which may have affected the results. Seventh, statistical assumptions for regression analyses (linearity, multicollinearity, and homoscedasticity) were not checked, which may influence the reliability of the reported associations. Eighth, this study was conducted at a single center, which may limit the generalizability of the results to other settings. Lastly, the physiology behind the findings could have been strengthened by incorporating biological markers of gut dysfunction, such as microbiome analysis or inflammatory biomarkers.

Future directions

Future studies need to overcome the limitations in the current study and address them with longitudinal and/or experimental studies to learn more about the directionality and causal nature of the findings of the GI-GBA and VM. The integration of biological biomarkers, such as stool microbiota profiles, measurement of gut permeability, or inflammatory cytokines, would help further explain the mechanisms. Additionally, it would be beneficial to assess the effectiveness of a gut-based approach (i.e., dietary modifications, probiotics, psychobiotics) in reducing the severity and frequency of VM symptoms. In future studies, additional information on diet, medication, comorbidities (e.g., diabetes, hypertension), and socioeconomic characteristics should also be obtained to collect detailed information regarding these confounders and support the adjustment in analysis to understand their impact on the apparent correlations. Generalizability to rural populations and the use of random sampling methodologies would be improved through sample expansion. Finally, the role of psychological comorbidities that cannot be assessed using the self-reported data may be better defined by including validated measures of their components in the assessment.

## Conclusions

This study found that GI symptoms and psychological comorbidities were associated with greater migraine-related disability and more severe vertigo symptoms, suggesting a potential role of gut-brain interactions in VM. Higher symptom burden was also associated with older age and female sex, demonstrating major demographic factors related to migraine and vestibular outcomes. These results highlight the significance of the multidimensional biopsychosocial guide in VM management, especially in an environment where GI and neurological disorders are commonly separated. A systematic evaluation of GI and psychological symptoms would enable targeted management and improved outcomes. Future research must utilize longitudinal study designs, validated psychological measures, and biological indicators of gut dysfunction to elucidate causal relationships and test gut-oriented therapeutics.

## Appendices

Appendix 

**Table 10:** Demographic Information Questionnaire

Items	Responses
Age	-
Gender	-
Marital Status	-
Educational Level	-
Occupation	-
Do you experience vertigo or dizziness episodes regularly?	-
Do you have any gastrointestinal (GI) issues? (e.g., bloating, constipation, reflux)	-
Have you been diagnosed with any psychological condition (e.g., anxiety, depression)?	-
Do you currently use any medication for migraine or GI issues?	
